# Severe acute pancreatitis in ICU: a 5-year audit

**DOI:** 10.1186/cc13387

**Published:** 2014-03-17

**Authors:** R Durrani, O Murphy, A Kibeida, G Fitzpatrick

**Affiliations:** 1 Tallaght Hospital & Trinity College Dublin, Ireland

## Introduction

Severe acute pancreatitis (SAP) is associated with significant mortality and morbidity. The objective of this study is to examine the profile, outcome and resource utilization for patients with SAP admitted to the ICU in a university teaching hospital over a 5-year period.

## Methods

A retrospective observational study was carried out of all patients admitted to the ICU from 1 January 2008 to 31 December 2012 with SAP. Data were collected from the ICU database (AcuBase), the medical records and the ICU clinical information system. Data collected included patient demographics, etiology of SAP, data for APACHE II, Imrie, Ranson and Acute Kidney Injury Network (AKIN) scores, and requirement for organ support. Outcomes recorded were length of stay, ICU mortality and hospital mortality. Cost of ICU care was calculated based on previously reported methodology.

## Results

Thirty-eight eligible patients were identified. Mean age was 51.4 years (range 24 to 86), 68% were male. The commonest etiologies were alcohol (53%) and gallstone pancreatitis (24%). The mean APACHE II score was 18.5 (IQR 14 to 23). Twenty-eight patients (74%) required mechanical ventilation, three of whom required high-frequency oscillation (all three survived). Twenty-two patients (58%) had evidence of an acute kidney injury on admission (AKIN criteria). Eighteen (47%) required renal replacement therapy and 60% required inotropes. The ICU mortality and the hospital mortality were 26%. There was no significant difference in age, APACHE II, Imrie, or Ranson scores between survivors and nonsurvivors. The median length of stay in the ICU was 11 days (IQR 5.25 to 28.5) and the median hospital stay was 45.4 days (IQR 22.25 to 104.5). Nine patients (24%) required multiple ICU admissions and the mortality was significantly higher in this group (*P *< 0.05, chi-square test). In total, 834 ICU bed-days were taken up by 38 patients. Based on a median cost for an ICU bed-day of €2,205 [1], the total cost of ICU care for these patients is estimated at €1,838,970 or almost €50,000 per patient.

## Conclusion

A hospital mortality rate of 26% is similar to that reported recently from a specialist unit in the UK [2] but less than the 42% reported in the UK in 2007 [1], suggesting some improvement in recent years. SAP is associated with prolonged ICU and hospital stay and significant resource utilization.
